# Comparison of US Hospital Charity Care Policies Before vs After Onset of the COVID-19 Pandemic

**DOI:** 10.1001/jamanetworkopen.2022.33629

**Published:** 2022-09-27

**Authors:** Christopher Goodman, Amber Flanigan, Janice C. Probst, Ge Bai

**Affiliations:** 1 Department of Internal Medicine, University of South Carolina School of Medicine, Columbia; 2 Department of Health Services Policy and Management, Arnold School of Public Health, University of South Carolina, Columbia; 3 Johns Hopkins Carey Business School, Baltimore, Maryland; 4 Johns Hopkins Bloomberg School of Public Health, Baltimore, Maryland

## Abstract

**Importance:**

Tax-exempt hospitals are required to provide charity care to maintain their tax-exempt status; charity care policies must be published online with clear eligibility criteria. Prior research has shown wide variability in charity care policy content; it is unknown how hospitals change their charity care policies over time.

**Objective:**

To examine changes to tax-exempt hospital charity care policies before vs after the COVID-19 pandemic.

**Design, Setting, and Participants:**

This cohort study used downloaded charity care policies from a geographically representative sample of 170 tax-exempt hospitals from December 1 to 31, 2019, and December 1 to 31, 2021, and categorized the policy changes made as more restrictive, more generous, indeterminate, minimal, or not updated.

**Exposures:**

Onset of the COVID-19 pandemic.

**Main Outcomes and Measures:**

The primary outcome was charity care policy content changes from 2019 to 2021. Also examined were the effects of hospital ownership type, state Medicaid expansion status, and hospital consolidation on policy changes.

**Results:**

In this sample of 170 hospitals, 151 published documents available for comparison. Among these hospitals, 127 (84.1%) updated their charity care policies and 77 (51.0%) made substantial changes, with 242 distinct policy changes to categories such as income eligibility cutoffs, asset limitations, and service exclusions. Although the majority of hospitals expanded charity care (47 [31.1%]), a sizable minority restricted charity care (12 [7.9%]). Medicaid expansion during the study period and hospital consolidations were not associated with expansion of charity care; the largest merger in this sample led to reduced charity care at all 4 hospitals involved.

**Conclusions and Relevance:**

Tax-exempt hospitals appear to have updated their policies with mostly positive changes during and after the onset of the COVID-19 pandemic; however, some hospitals restricted charity care in 2021 documents. Unpublicized or vague eligibility criteria may limit patients’ understanding of charity care policies and conceal the full extent of charity care policy changes over time. Policy makers should consider requiring greater transparency and simplification for hospital charity care policies to ensure adequate access to care for uninsured and underinsured patients.

## Key Points

**Question:**

Did hospital charity care policies change after the onset of the COVID-19 pandemic?

**Findings:**

In this cohort study of 170 tax-exempt hospitals, 151 had charity care policies available in 2019 and 2021 for comparison, and 127 (84.1%) updated their charity care policies. Hospitals preferentially expanded charity care (47 [31.1%] vs 12 [7.9%] with restrictive changes); however, unpublicized and vague eligibility criteria were common, and Medicaid expansion and hospital consolidations were not associated with expansion of charity care.

**Meaning:**

These findings suggest that between 2019 and 2021, tax-exempt hospitals updated their charity care policies with mostly positive changes; however, restrictions in charity care and unclear eligibility criteria are common and deserve regulatory attention.

## Teaser

This cohort study compares charity care policies in a representative sample of large US hospitals between December 2019 and December 2021.

## Introduction

Tax-exempt hospitals are required by the Affordable Care Act to establish and publicize written charity care policies (*financial assistance policies* in regulatory terminology) that enumerate eligibility criteria and covered services to maintain their tax-exempt status.^1^ Charity care provided by these hospitals, especially safety-net hospitals that play a major role in serving low-income communities, is a key justification for these hospitals’ tax exempt status and an important policy tool curbing the financial burden of increasing health care costs for uninsured and underinsured patients.^2^ Despite coverage gains from the Affordable Care Act, 13.9% of nonelderly adults in 2020 were uninsured and potentially eligible for charity care.^3^ Charity care policies contain certain types of content specified by federal regulations; however, thresholds for eligibility and the scope of services provided are determined by individual hospitals. In 2019, Goodman et al^4^ examined the content of charity care policies and identified common eligibility criteria with wide variability in additional eligibility restrictions, charges, and service exclusions.

Since that original analysis, the hospital industry has experienced major financial disruption associated with the COVID-19 pandemic. How this disruption would affect charity care was uncertain, with some evidence^5^ suggesting that hospitals were expanding patient financial assistance, whereas other investigators^6^ found that the top 15 systems reduced charity spending. Therefore, we revisited the 2019 sample of large hospitals and compared charity care policies obtained 2 years apart (December 1 to 31, 2019, vs December 1 to 31, 2021). Based on prior observations of charity care policies, we hypothesized that charity care policies would be more generous in 2021 than in 2019.

## Methods

### Study Cohort

This cohort study followed the Strengthening the Reporting of Observational Studies in Epidemiology (STROBE) reporting guideline. In 2019, Goodman et al^4^ developed a sample of large, acute care hospitals using the following algorithm:

1. The top 50 nonprofit hospitals by gross receipts as obtained through GuideStar, which provides comprehensive information about nonprofit organizations and is available online.^7^
2. The next 50 nonprofit hospitals by gross receipts from the same source, excluding hospitals in states for which 2 hospitals were already present after using criterion 1.
3. The largest nonprofit or government hospital by bed number in each state, according to the American Hospital Directory online.^8^
4. The largest nonprofit or government hospital in 2 separate hospital referral regions in a given state not represented by criteria 1 to 3 (defined by the Dartmouth Atlas Project^9^).

We chose larger, tax-exempt hospitals to select for safety-net hospitals, which account for a disproportionate share of care to uninsured patients and those with Medicaid coverage who are more likely to depend on charity care services from hospitals. If an organization identified through GuideStar was a multihospital system, we chose the system’s largest hospital. Using this process, we identified a sample of 170 hospitals and downloaded their charity care policies in December 2019 (eTable 1 in the Supplement). Among them, 22 hospitals were government hospitals (state or local government), and the remaining were nonprofit hospitals.^10^ We included government hospitals because they are tax-exempt and subject to Section 501(r) of the Internal Revenue Code.^1^ Hospitals are often part of multihospital systems, and charity care policies often apply across the entire organization. We estimate that based on system affiliations, our sample of charity care policies represents charity care at approximately 1200 hospitals, or one-fifth of all acute care US hospitals.

In December 2021, we replicated the process of downloading charity care policies for the same hospitals. Nineteen hospitals did not have charity care policies available for analysis for both years and were excluded: policies at 7 hospitals were downloaded incorrectly in 2019, and 12 hospitals did not publish full charity care policies in at least 1 year. The final sample for analysis contained 151 hospitals.

### Coding of Charity Care Policies

Policies were first coded on whether an update occurred between the documents downloaded in 2019 and 2021. Next, if updates were evident, significant criteria changes were noted. Categories of criteria changes were developed iteratively through the process of reviewing policies. Each criterion change was individually coded as more generous, more restrictive, or indeterminate. We considered any policy change that expanded access to financial assistance to be more generous and any policy change that restricted access to be more restrictive. For example, an increase in the income cutoff for free care from 150% to 200% of the federal poverty level would be more generous. If the direction of change was uncertain owing to lack of specificity in policy language, the category was coded as indeterminate. For example, a new policy may specify a duration of coverage for 6 months, and the old policy may not specify duration. In this scenario, the change would be coded as indeterminate because it is unknown whether the new specification is more benefical, more restrictive, or unchanged. Policy changes related to grammar or formatting or that generally did not appear to substantially affect access were not coded individually.

Last, assessments of criteria changes were combined to give an overall assessment of more generous, more restrictive, indeterminate, or minimal change. Policies were coded as minimal change if changes did not substantially affect charity care (eg, formatting changes only). Hospitals with conflicting directionality in changes to criteria in which it was difficult to make an overall assessment were coded as indeterminate.

Coding of policies was performed by 2 authors (C.G. and A.F.). The first author (C.G.) conducted the initial analysis of all hospital charity care policies. The second author (A.F.) audited a sample of hospitals (20% of hospitals with significant changes), and the discussion informed reanalysis of the entire sample. The collaborative coding process identified 23 categories of policy changes (Table 1).

**Table 1.** Categories of Charity Care Policy Changes, December 2021 vs December 2019

Category	Definitions
Income (free)	Income cutoff for free care, usually by percentage of federal poverty level
Income (discount)	Income cutoff for discounted care, usually by percentage of federal poverty level
Assets	Assets used for eligibility determination
Residency	Location of residence for eligibility
Presumptive	Eligibility criteria that automatically make someone eligible for charity care, usually resulting in an abbreviated application process (eg, homelessness or Medicaid status)
Family and household	Definitions of family or household used in examining income and assets
Underinsured	General eligibility for charity care despite existing insurance
Network	Coverage for out-of-network care for insured patients
Cost share	Coverage of insurance copayments, deductibles, and coinsurance
Other insurance	Difficult to categorize content related to unique insurance situations
Medicaid	Coverage of cost sharing for patients with Medicaid coverage
Duration	Duration of coverage once approved
Services	Types of services covered
Retroactive	Duration of coverage for past services once approved
Catastrophic	Policy content related to discounts for very high balances in relation to income
Minimum balance	Minimum balances to which charity care applies or at which hospitals will take legal action to collect
Discount amount	Changes to amount of discount applied
Self-pay discount	Standard discounts for all patients regardless of charity care eligibility
Fees	Expected payments related to charity care services
COVID-19 clause	Coverage directly related to the COVID-19 pandemic
Collections	Process for collecting payment
Discount tiers	Use of tiered coverage based on eligibility criteria
Third-party tool	Use of a third-party tool for eligibility determinations

### Statistical Analysis

Our analysis compared hospitals based on 3 factors influencing charity care as suggested by prior literature: (1) whether the hospital is in a state that expanded Medicaid eligibility before or during the analysis period, (2) whether ownership changed owing to a merger or acquisition, and (3) whether the hospital is a nonprofit or a government hospital.^10,11^ Merger and acquisition status was determined by comparing identifying information on charity care policy documents. Statistical analysis used the Fisher exact test to address small cell sizes in some categories, with α = .05 indicating significance. Given the small number of observations, particularly when the sample is subdivided by category (eg, only 9 of 151 studied systems experienced a merger or an acquisition), multivariable analysis was not attempted. Analyses were performed using Stata, version 17.0 (StataCorp LLC).

## Results

### Changes to Charity Care Policies

**Figure 1.**
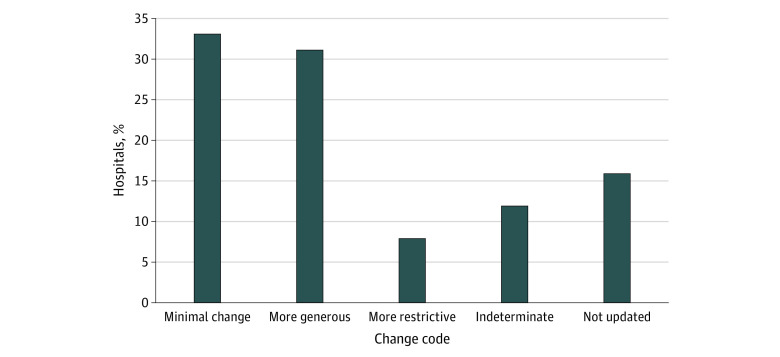
Overall Change in Charity Care Policy, December 2021 vs December 2019 The sample contains 151 hospitals. Definitions of change codes are given in the Coding of Charity Care Policies subsection of the Methods section.

**Figure 2.**
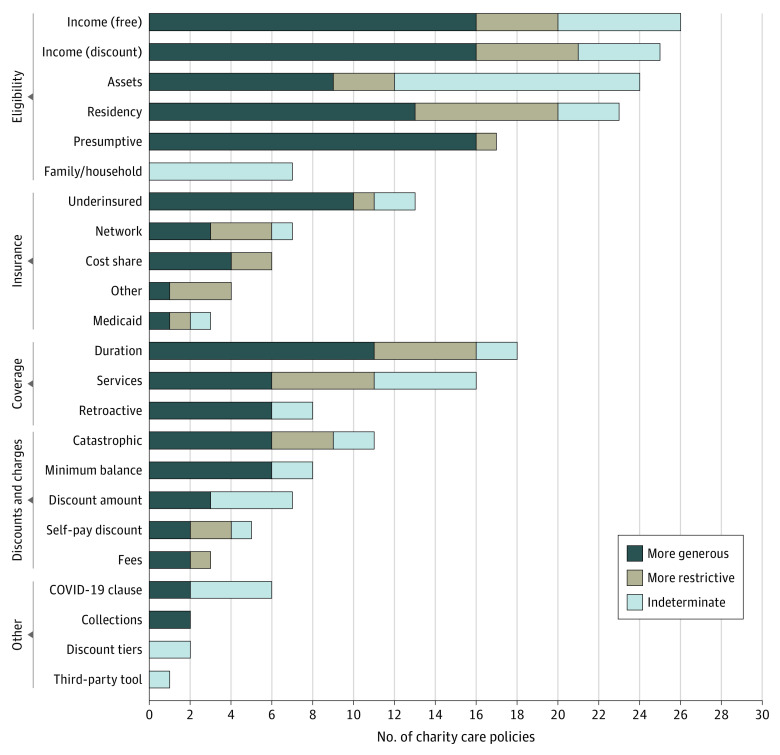
Number of Charity Care Policy Changes by Type December 2021 vs December 2019

Charity care policies were available for 151 hospitals for analysis. Across the 2-year period, 127 hospitals (84.1%) updated their policies (Figure 1), and 77 (51.0%) made substantial changes. Fifty-nine hospitals adopted either distinctively more generous or more restrictive policies. Among them, 47 hospitals (31.1%) made their policies more generous, and 12 hospitals (7.9%) made their policies more restrictive. We identified 242 distinct policy changes: 135 were more generous, 46 were more restrictive, and 61 were indeterminate (Figure 2). A complete list of charity care policy changes is provided in eTable 2 in the Supplement.

Hospitals mostly increased their charity care generosity across all criteria, most frequently by expanding eligibility criteria. Income cutoffs for free (n = 26) and discounted (n = 25) care were the criteria most frequently changed; 16 hospitals increased their income cutoffs for each criterion. Presumptive eligibility was changed in 17 policies and nearly always expanded (n = 16). Presumptive eligibility involves the use of certain patient characteristics that suggest financial need, such as homelessness or lack of employment, to shorten the application process. Examples of presumptive eligibility expansion included adding current Medicaid coverage and incarceration as criteria.

Assets involved in eligibility determinations were changed frequently (n = 24); however, half of the changes were indeterminate owing to nonspecific language. The most common scenario was that the 2019 policy made a generic statement about examining assets and the 2021 policy specified asset cutoffs, which is not clearly better or worse given that the thresholds for assets under the old policy were not specified. When specified, new cutoffs for total assets were wide ranging ($20 000 to $1 million). A few hospitals made simple changes to language about assets that were straightforward to assess; however, changes were more often complex. For example, 1 hospital stated in its 2019 policy that “substantial liquid assets” might be an exclusion without specifying cutoffs. In 2021, its updated policy made specific changes to include property, exclude certain forms of retirement investments, and specify a total asset cutoff of $20 000.

Residency requirements were restricted more often than any other individual criteria change (n = 7); hospitals either narrowed eligibility to the service area of the hospital (a county-based area in the immediate vicinity of the hospital) or restricted eligibility based on immigration status. Immigration status was the most common focus of residency requirement changes. One hospital changed its eligibility requirement from US citizens and residents to anyone who “lives in the US,” which we considered an expansion of eligibility. Another hospital added a new exclusion of any “foreign resident,” which we assessed as restrictive. One hospital took the additional step of specifying how long a person had to live in the US (18 months) before they could be considered eligible for charity care.

Services covered were changed at 16 hospitals, and several unusual exclusions were added. One hospital added a new exclusion for any care related to self-harm. Another hospital excluded care provided while the patient is in the custody of law enforcement. One hospital made only a single change to its policy to exclude birth control from charity care. Most changes to covered services involved adding or removing coverage of certain professional services (eg, outpatient care or urology specialty care). In determining changes to covered services, we avoided including changes that appeared to be related to mergers and acquisitions and changes to clinician affiliations. Such changes could be considered new locations of care and potentially new services, but they are unrelated to decisions on what constitutes charity care itself.

General changes regarding the inclusion of underinsured individuals were common (n = 10); however, hospitals rarely defined the term *underinsured*, and specific changes to coverage of cost sharing (ie, copayments, coinsurances, and deductibles) and out-of-network charges were mixed. Some hospitals expanded coverage (n = 7) and some restricted coverage (n = 5). Among the hospitals that restricted coverage, several hospitals cited contractual obligations to insurers to justify the change. Three hospitals added language that specifically disallowed the patient option of opting for charity care coverage rather than using health insurance.

Six hospitals added what we termed *COVID-19 clauses*. These additions usually stated that the hospital could make ad hoc policy changes to charity care during public health emergencies. It is unclear what effects these policy changes had during the COVID-19 pandemic. Only 2 hospitals wrote such policies to clearly expand coverage of services related to COVID-19 testing and treatment.

### Association of Health System Factors With Charity Care

Hospitals that experienced mergers and/or acquisitions between 2019 and 2021 (n = 9) were identified based on changes to policy documents; therefore, they were all labeled as having updated policies in our sample. Changes to their charity care policies were more likely to have a definitive direction (4 [44.4%] more generous and 4 [44.4%] more restrictive) than to be minimal (0) or indeterminate (1 [11.1%]) (*P* = .002) (Table 2). Four hospitals represented 2 hospital systems in 2019 that merged during the study period; the resulting single charity care policy for all 4 hospitals was more restrictive than the 2 prior policies. Furthermore, the new shared charity care policy resulted in restrictions to 12 policy categories, which is 26.1% of all 46 restrictive changes in our sample.

**Table 2.** Mergers and Acquisitions, State Medicaid Expansion, and the Overall Change in Charity Care Policy, December 2021 vs December 2019

Change	Total, No. (%)	Mergers and acquisition, No. (%)		*P* value	Medicaid expansion state, No. (%)^a^		*P* value
		Yes	No		Yes	No	
Update status							
Not updated	24 (16)	0	24 (16.9)	.36	15 (12.4)	9 (30.0)	.03
Updated	127 (84)	9 (100)	118 (83.1)		106 (87.6)	21 (70.0)	
Total	151 (100)	9 (100)	142 (100)		121 (100)	30 (100)	
Type of change (updated hospitals only)							
More generous	47 (37)	4 (44.4)	43 (36.4)	.002	35 (33.0)	12 (57.1)	.18
More restrictive	12 (9)	4 (44.4)	8 (6.8)		10 (9.4)	2 (9.5)	
Indeterminate	18 (14)	1 (11.1)	17 (14.4)		17 (16.0)	1 (4.8)	
Minimal or none	50 (39)	0	50 (42.4)		44 (41.5)	6 (28.6)	
Total	127 (100)	9 (100)	118 (100)		106 (100)	21 (100)	

^a^ Nine hospitals were in 4 states that expanded Medicaid from 2019 to 2021 (Idaho, Nebraska, Oklahoma, and Utah; Missouri hospitals were considered nonexpansion, given the litigation that has halted expansion). Seven of these hospitals updated their plans; however, there were no significant differences in update status (*P* = .64) or directionality of change (*P* = .22) between hospitals in “new” expansion states and those that either never expanded Medicaid or were expansion states in 2019.

Hospitals located in Medicaid expansion states were more likely than other hospitals to have updated plans in 2021 (106 of 121 [87.6%] vs 21 of 30 [70.0%]; *P* = .03, Fisher exact test); however, the type of change was not statistically significant (eg, more generous, 35 of 106 [33.0%] vs 12 of 21 [57.0%]; *P* = .18 for overall comparison). Among hospitals located in Medicaid expansion states, 9 were in 4 states that expanded Medicaid from 2019 to 2021. Seven of these hospitals updated their plans, similar to the proportion among all other hospitals (77.8% vs 84.5%; *P* = .64), and the direction of change was similar as well (2 were more generous, 2 were more restrictive, and 3 were indeterminate; *P* = .22). Hospital ownership type (nonprofit or government) was not associated with update status or direction of change.

## Discussion

Many people rely on hospital charity care to relieve the financial burden of health care costs; this reliance has been especially important during the COVID-19 pandemic given the related increases in job loss and disability.^12^ We found that between December 2019 and December 2021, almost one-third of 151 large, tax-exempt hospitals improved their charity care policies, commonly through expansion of eligibility criteria. Specific policy changes mostly involved the 6 criteria we identified in our prior analysis as common to most charity care policies: income cutoffs for free and discounted care, residency status, presumptive eligibility, duration of coverage, and underinsured eligibility.^4^ These expansions are remarkable given the lack of federal requirements to do so. However, there are reasons for concern despite the generally positive changes.

First, despite the extraordinary circumstances of the COVID-19 pandemic and historic insurance coverage gains through the Affordable Care Act, 7.9% of studied hospitals unambiguously restricted charity care through eligibility requirements and coverage changes. Residency requirements were the most frequently restricted, often by limiting access based on immigration status. Some hospitals added unusual service restrictions such as exclusion of coverage for services while in the custody of law enforcement and exclusion of birth control.

Second, charity care policies continue to use vague language, especially for eligibility criteria such as assets, which limits patients’ understanding of charity care policies and may conceal policy changes over time. Third-party tools for eligibility determination are common and may further limit transparency of eligibility requirements. In addition, charity care policies of 12 hospitals in our sample were not readily available in at least 1 year in 2019 and 2021. Inadequate descriptions of eligibility criteria and inaccessible policies that are potentially noncompliant with Internal Revenue Service regulations make it challenging for patients to understand their charity care options.^1^

Third, although underinsured eligibility expanded among hospitals in our sample, when hospitals addressed specific insurance scenarios common to underinsured status (eg, cost-sharing and out-of-network services), the results were mixed. Among hospitals that restricted coverage related to cost sharing, some alluded to contracts with insurers that limit charity care coverage, which conflicts with the spirit of the community benefit requirements of tax-exempt hospitals. The impact of these types of changes on insured patients is complex and difficult to fully ascertain, given geographic variation in health care charges and insurance networks.^13^ Nevertheless, given the increasing burden of financial costs even for insured patients, expansion of charity coverage for insured patients is clearly desirable, and hospital coverage decisions vary.

Last, it might seem logical that economies of scale for hospital consolidation could lead to expansion of charity care policies; similarly, Medicaid expansion reduces the uninsured population and could cause hospitals to adopt more generous charity care policies. However, the results in our analysis do not provide supportive evidence, which is in line with prior research.^2,14^

### Limitations

This study has several limitations. First, because we relied on information disclosed by hospitals, our findings may be inaccurate to the extent that documents available online may have become outdated or otherwise may not reflect actual practice. Second, although we reviewed the policies multiple times to ensure accuracy, it is possible that some policy changes were miscategorized during the review process, especially for complex policies with major rearrangements. Third, the vague language used by some hospitals limited our ability to understand the association between policy content and access to charity care. For example, assets are often mentioned as part of eligibility criteria without specifying cutoffs, and the use of third-party tools for financial eligibility determination are common. Last, the results of our study, represented by large hospitals, may not be generalizable to smaller hospitals.

## Conclusions

In this cohort study, we found that many large, tax-exempt hospitals changed their policies during the COVID-19 pandemic, often making expansions for the benefit of their communities. However, some hospitals restricted access to charity care through complex eligibility requirements and coverage changes. Nonspecific language is common in charity care policies, which limited our analysis and may conceal additional changes over time. Our findings suggest that greater transparency and simplification in charity care policies—especially for eligibility criteria—are needed to ensure adequate access to charity care.

## Notes

Supplement. **eTable 1.** List of Hospitals in Our Sample Organized by State (n = 170) **eTable 2.** Changes in Charity Care Policy, December 2019 vs December 2021
