# Supplementary material for: Comparison of US Hospital Charity Care Policies Before vs After Onset of the COVID-19 Pandemic
Source: JAMA Netw Open. 2022 Sep 27;5(9):e2233629. doi: 10.1001/jamanetworkopen.2022.33629 (PMC9516315; doi:10.1001/jamanetworkopen.2022.33629)
Supplement: Supplement. — eTable 1. List of Hospitals in Our Sample Organized by State (n = 170) eTable 2. Changes in Charity Care Policy, December 2019 vs December 2021 [file jamanetwopen-e2233629-s001.pdf]

Supplementary Online Content

Goodman C, Flanigan A, Probst JC, Bai G. Comparison of US hospital charity care policies before vs after onset of the COVID-19 pandemic. *JAMA Netw Open*. 2022;5(9):e2233629. doi:10.1001/jamanetworkopen.2022.33629

eTable 1. List of Hospitals in Our Sample Organized by State (n = 170)
eTable 2. Changes in Charity Care Policy, December 2019 vs December 2021

This supplementary material has been provided by the authors to give readers additional information about their work.

© 2022 Goodman C et al. *JAMA Network Open*.

eTable 1. List of Hospitals in Our Sample Organized by State (n = 170)

State	Hospital	City	Beds	Type
Alabama	UAB hospital	Birmingham	1201	Government - State
	Hunstville	Huntsville	903	Government - Hospital District or Authority
	Mobile Infirmary	Mobile	689	Voluntary non-profit - Private
Alaska	Providence	Anchorage	379	Voluntary non-profit - Church
Arizona	Banner - University Medical Center Phoenix	Phoenix	733	Voluntary non-profit - Other
	Banner Desert	Mesa	615	Voluntary non-profit - Private
	Banner - University Medical Center Tucson	Tucson	479	Voluntary non-profit - Private
Arkansas	Baptist Health Medical Center - Little Rock	Little Rock	814	Voluntary non-profit - Private
	Mercy Hospital Fort Smith	Fort Smith	336	Voluntary non-profit - Private
	St. Bernards Medical Center	Jonesboro	355	Voluntary non-profit - Private
California	Providence - Little Company of Mary Medical Center - Torrance	Torrance	442	Voluntary non-profit - Church
	Dignity - Northridge Hospital	Northridge	424	Voluntary non-profit - Private
	Stanford	Stanford	477	Voluntary non-profit - Private
	Cedars-Sinai	Los Angeles	879	Voluntary non-profit - Other
	Scripps Mercy Hospital San Diego	San Diego	523	Voluntary non-profit - Private
	Sutter Bay - California Pacific - Pacific Campus	San Francisco	480	Voluntary non-profit - Private

© 2022 Goodman C et al. *JAMA Network Open*.

	Sutter Valley - Sutter Medical Center	Sacramento	523	Voluntary non-profit - Private
	Community Regional Medical Center	Fresno	909	Voluntary non-profit - Private
	Kaiser Permanente - Fontana	Fontana	626	Voluntary non-profit - Private
Colorado	Poudre Valley	Fort Collins	258	Voluntary non-profit - Private
	UC-Health University	Denver	655	Government - Hospital District or Authority
	UC-Health Central	Colorado Springs	497	Government - Local
	North Colorado - Banner	Greeley	225	Voluntary non-profit - Private
Connecticut	Hartford	Hartford	938	Voluntary non-profit - Private
	Yale	New Haven	1540	Voluntary non-profit - Private
	Bridgeport	Bridgeport	359	Voluntary non-profit - Private
Delaware	Christiana Care	Newark	1128	Voluntary non-profit - Private
District of Columbia	Medstar - Georgetown	Washington, DC	396	Voluntary non-profit - Other
	Medstar - Washington	Washington, DC	752	Voluntary non-profit - Other
Florida	UF Health Shands	Gainesville	871	Voluntary non-profit - Private
	Florida Hospital Orlando	Orlando	2635	Voluntary non-profit - Private
	Baptist Medical Center Jacksonville	Jacksonville	903	Voluntary non-profit - Private
	Jackson Memorial	Miami	1707	Government - Hospital District or Authority
Georgia	Northside	Atlanta	524	Voluntary non-profit - Private
	Kennestone	Atlanta	662	Government - Hospital District or Authority
	Grady	Atlanta	961	Voluntary non-profit - Private

© 2022 Goodman C et al. *JAMA Network Open*.

	Medical Center Navicent Health	Macon	597	Government - Hospital District or Authority
	University Hospital	Augusta	560	Government - Hospital District or Authority
Hawaii	Hawaii Pacific Health - Kapiolani Medical Center	Honolulu	207	Voluntary non-profit - Private
	Queen's Medical Center	Honolulu	357	Voluntary non-profit - Private
Idaho	Saint Luke's	Boise	578	Voluntary non-profit - Private
Illinois	Southern Illinois - Memorial Hospital	Carbondale	159	Voluntary non-profit - Private
	OSF St. Francis	Peoria	648	Voluntary non-profit - Private
	Rush University Medical Center	Chicago	675	Voluntary non-profit - Private
	Northshore - Evanston	Evanston	789	Voluntary non-profit - Other
	Northwestern Memorial Hospital	Chicago	900	Voluntary non-profit - Private
	Carle Foundation	Urbana	411	Voluntary non-profit - Private
	Presence St. Joseph	Joliet	466	Voluntary non-profit - Church
Indiana	IU Health University	Indianapolis	1286	Voluntary non-profit - Private
	Deaconess Midtown	Evansville	483	Voluntary non-profit - Private
	Parkview Regional Medical Center	Fort Wayne	710	Voluntary non-profit - Private
	Methodist Hospitals - North Lake	Gary	498	Voluntary non-profit - Private
Iowa	University of Iowa Hospitals and Clinics	Iowa City	728	Government - State
	Mercy Medical Center	Des Moines	576	Voluntary non-profit - Private
	Unity Point Health - Cedar	Cedar Rapids	344	Voluntary non-profit - Private

© 2022 Goodman C et al. *JAMA Network Open*.

	Rapids - St. Luke's			
Kansas	University of Kansas	Kansas City	768	Government - Hospital District or Authority
	Stormont Vail	Topeka	449	Voluntary non-profit - Private
	Via Christi Saint Francis	Wichita	658	Voluntary non-profit - Private
Kentucky	Norton	Louisville	1364	Government - Local
	Saint Elizabeth	Edgewood	487	Voluntary non-profit - Church
	Owensboro Health Regional	Owensboro	477	Voluntary non-profit - Private
Louisiana	Ochsner Medical Center	New Orleans	961	Voluntary non-profit - Private
	Our Lady of the Lake	Baton Rouge	834	Voluntary non-profit - Church
	Willis Knighton	Shreveport	665	Voluntary non-profit - Private
	Saint Francis Medical Center Downtown	Monroe	507	Voluntary non-profit - Private
Maine	Northern Light Eastern Maine Medical Center	Bangor	349	Voluntary non-profit - Private
	Maine Medical Center	Portland	637	Voluntary non-profit - Private
Maryland	The Johns Hopkins Hospital	Baltimore	997	Voluntary non-profit - Private
	University of Maryland Medical Center	Baltimore	802	Voluntary non-profit - Private
	Peninsula Regional Medical Center	Salisbury	281	Voluntary non-profit - Private
	Holy Cross	Silver Spring	455	Voluntary non-profit - Church
Massachusetts	Beth Israel Deaconess	Boston	686	Voluntary non-profit - Private
	Massachusetts General	Boston	1011	Voluntary non-profit - Private
	Baystate Medical Center	Springfield	720	Voluntary non-profit - Private
	UMass Memorial Medical Center -	Worcester	625	Voluntary non-profit - Private

© 2022 Goodman C et al. *JAMA Network Open*.

	University Campus			
Michigan	Beaumont Hospital, Royal Oak	Royal Oak	1089	Voluntary non-profit - Private
	Spectrum Health Butterworth	Grand Rapids	1106	Voluntary non-profit - Private
	University of Michigan Hospitals and Health Centers	Ann Arbor	998	Voluntary non-profit - Private
	Henry Ford	Detroit	682	Voluntary non-profit - Private
Minnesota	Mayo Clinic - St. Mary's Campus	Rochester	1297	Voluntary non-profit - Church
	University of Minnesota - Fairview Health	Minneapolis	829	Voluntary non-profit - Private
	Saint Cloud	St. Cloud	487	Voluntary non-profit - Private
Mississippi	North Mississippi Medical Center	Tupelo	630	Voluntary non-profit - Private
	Forrest General	Hattiesburg	470	Government - Local
Missouri	Cox Hospital	Springfield	670	Voluntary non-profit - Private
	Barnes-Jewish	St. Louis	1303	Voluntary non-profit - Other
	University of Missouri	Columbia	587	Government - State
	Freeman West	Joplin	391	Voluntary non-profit - Private
Montana	Benefis Healthcare	Great Falls	478	Voluntary non-profit - Private
	Kalispell Regional Medical Center	Kalispell	288	Voluntary non-profit - Private
	Billings Clinic Hospital	Billings	283	Voluntary non-profit - Private
Nebraska	Bryan Medical Center	Lincoln	559	Voluntary non-profit - Private
	The Nebraska Medical Center	Omaha	532	Voluntary non-profit - Private
Nevada	Renown Regional Medical Center	Reno	619	Voluntary non-profit - Private

© 2022 Goodman C et al. *JAMA Network Open*.

	Saint Rose Dominican Hospital - Siena Campus	Henderson	326	Voluntary non-profit - Private
New Hampshire	Dartmouth-Hitchcock	Lebanon	402	Voluntary non-profit - Private
	Catholic Medical Center	Manchester	258	Voluntary non-profit - Private
New Jersey	Hackensack University Medical Center	Hackensack	711	Voluntary non-profit - Private
	Robert Wood Johnson University Hospital New Brunswick	New Brunswick	610	Voluntary non-profit - Private
	Saint Joseph's University Medical Center	Paterson	697	Voluntary non-profit - Church
	New Bridge Medical Center	Paramus	1013	Government - Local
New Mexico	Presbyterian	Albequerque	771	Voluntary non-profit - Private
New York	New York-Presbyterian	New York City	2410	Voluntary non-profit - Private
	Montefiore Hospital - Moses Campus	New York City	1520	Voluntary non-profit - Private
	Long Island Jewish Medical Center	New York City	1547	Voluntary non-profit - Private
	The Mount Sinai Hospital	New York City	1103	Voluntary non-profit - Private
	North Shore University	Manhasset	1040	Voluntary non-profit - Private
	Buffalo General	Buffalo	1068	Voluntary non-profit - Private
	Strong Memorial	Rochester	823	Voluntary non-profit - Private
North Carolina	Wake Forest University Baptist Medical Center	Winston Salem	873	Voluntary non-profit - Private

© 2022 Goodman C et al. *JAMA Network Open*.

	Novant Health Forsyth Medical Center	Winston Salem	914	Voluntary non-profit - Other
	Carolinas Medical Center	Charlotte	1229	Government - Hospital District or Authority
	Duke University	Durham	954	Voluntary non-profit - Private
	Vidant Medical Center	Greenville	909	Voluntary non-profit - Private
North Dakota	Trinity	Minot	493	Voluntary non-profit - Private
	Sanford Bismarck Medical Center	Bismarck	237	Voluntary non-profit - Private
	Altru	Grand Forks	299	Government - Local
Ohio	OhioHealth Riverside Methodist	Columbus	716	Voluntary non-profit - Private
	ProMedica Toledo	Toledo	720	Voluntary non-profit - Private
	The Cleveland Clinic	Cleveland	1283	Voluntary non-profit - Private
	Miami Valley	Dayton	810	Voluntary non-profit - Private
	Summa Akron City	Akron	635	Voluntary non-profit - Private
Oklahoma	Oklahoma University Medical Center	Oklahoma City	711	Voluntary non-profit - Private
	Saint Francis	Tulsa	858	Voluntary non-profit - Private
	Comanche County Memorial	Lawton	250	Government - Hospital District or Authority
Oregon	Providence Saint Vincent Medical Center	Portland	474	Voluntary non-profit - Private
	Salem	Salem	423	Voluntary non-profit - Other
	Asante Rogue Regional Medical Center	Medford	340	Voluntary non-profit - Private
	Oregon Health and Science University Hospital	Portland	556	Voluntary non-profit - Other

© 2022 Goodman C et al. *JAMA Network Open*.

	Peacehealth Sacred Heart Center at Riverbend	Springfield	379	Voluntary non-profit - Private
Pennsylvania	Lehigh Valley Hospital - Cedar Crest	Allentown	897	Voluntary non-profit - Private
	UPMC Pinnacle Harrisburg	Harrisburg	640	Voluntary non-profit - Other
	UPMC Presbyterian	Pittsburg	1483	Voluntary non-profit - Private
	Thomas Jefferson University Hospital	Philadelphia	899	Voluntary non-profit - Private
	Reading	West Reading	678	Voluntary non-profit - Private
Rhode Island	Rhode Island	Providence	693	Voluntary non-profit - Private
South Carolina	McLeod Regional Medical Center	Florence	509	Voluntary non-profit - Private
	Greenville Memorial	Greenville	814	Voluntary non-profit - Private
	Palmetto Health Richland	Columbia	721	Voluntary non-profit - Private
	Medical University of South Carolina	Charleston	773	Government - State
South Dakota	Avera McKennan University Hospital	Sioux Falls	636	Voluntary non-profit - Private
	Regional Health Rapid City	Rapid City	411	Voluntary non-profit - Private
Tennessee	Methodist University	Memphis	1383	Voluntary non-profit - Church
	Vanderbilt University	Nashville	993	Voluntary non-profit - Private
	Jackson-Madison County General	Jackson	690	Government - Hospital District or Authority
Texas	Seton Medical Center	Austin	410	Voluntary non-profit - Private

© 2022 Goodman C et al. *JAMA Network Open*.

	Baylor University Medical Center at Dallas	Dallas	854	Voluntary non-profit - Church
	Methodist	San Antonio	1576	Proprietary (50% HCA)
	Memorial Hermann Southwest	Houston	1411	Voluntary non-profit - Private
	Texas Health Harris Methodist Fort Worth	Fort Worth	666	Voluntary non-profit - Church
Utah	University of Utah Health Care	Salt Lake city	574	Government - State
	McKay-Dee - Ogden	Ogden	312	Voluntary non-profit - Private
	Utah Valley - Provo	Provo	359	Voluntary non-profit - Private
Vermont	University of Vermont	Burlington	450	Voluntary non-profit - Other
Virginia	Sentara Norfolk General	Norfolk	524	Voluntary non-profit - Other
	Carilion Roanoke Memorial	Roanoke	703	Voluntary non-profit - Private
	Inova Fairfax	Falls Church	334	Voluntary non-profit - Private
	Centra Lynchburg General	Lynchburg	703	Voluntary non-profit - Private
Washington	Swedish First Hill	Seattle	701	Voluntary non-profit - Private
	Saint Joseph Medical Center	Tacoma	366	Voluntary non-profit - Church
	Providence Sacred Heart Medical Center	Spokane	640	Voluntary non-profit - Church
West Virginia	Charleston Area Medical Center	Charleston	868	Voluntary non-profit - Private
	J.W. Ruby Memorial Hospital	Morgantown	621	Voluntary non-profit - Private
	Saint Mary's	Huntington	379	Voluntary non-profit - Church

© 2022 Goodman C et al. *JAMA Network Open*.

Wisconsin	Froedtert and the Medical College of Wisconsin	Milwaukee	553	Voluntary non-profit - Private
	Aurora Saint Luke's Medical Center	Milwaukee	919	Voluntary non-profit - Private
	Gundersen Lutheran Medical Center	La Crosse	268	Voluntary non-profit - Private
	University Hospital	Madison	609	Government - Hospital District or Authority
Wyoming	Cheyenne Regional Medical Center	Cheyenne	222	Voluntary non-profit - Other
	Wyoming Medical Center	Casper	163	Government - Local

© 2022 Goodman C et al. *JAMA Network Open*.

eTable 2. Changes in Charity Care Policy, December 2019 vs December 2021

More generous	More restrictive	Indeterminate
Increases free cutoff from 100% FPL to 200% FPL; Increases presumptive eligibility (including removal of requirement of $6,500 minimal balance for insured patients)		
Increases discount care income cutoff from 300% FPL to 350% FPL; Adds retroactive for 6 months; Adds automatic discount of 35% regardless of charity care eligibility; Specifies discount percentage calculation; Reduces minimum balance eligibility from $25 to $10; Expands presumptive eligibility		Income cutoff for free care no longer specified
		Adds clause allowing for changes to eligibility determinations during public health emergencies
Extensive addition of presumptive eligibility	Adds residency requirement to live in service area of hospital; Adds exclusion of "foreign	Specifies retroactive for 12 months; Removes language in assets that first $10k in assets (plus

© 2022 Goodman C et al. *JAMA Network Open*.

	residents;" Decreases income cutoff for free care from 250% FPL to 200% FPL; Decreases discount cutoff from 500% FPL to 400% FPL (also technical change to discount calculation)	50% of next $10k) would be excluded from consideration; Hardship discount section removed but appears it may be in a separate policy; Adds required minimum balance of $10
		Eligible services clarified to include “hospital and its affiliated clinics,” however unclear whether clinic services were included before
Adds coverage of Medicaid denials; Increases income cutoff for free care from 200% FPL to 400% FPL; Increases income cutoff for discounted care from 450% FPL to 600% FPL	Removes automatic self-pay discounts section; Lowers income range for eligibility for catastrophic coverage from 600% FPL to 450% FPL	
		Excludes out of network services
	Excludes "office visits" including telehealth by Hartford Medical Group providers	
Expands residency eligibility from “US citizens and residents” to simply “live in the		

© 2022 Goodman C et al. *JAMA Network Open*.

US;” Adds eligibility for insured patients to discounted care income tier		
Expands residency eligibility from “US citizens and residents” to “live in the US;” Adds eligibility for insured patients to discounted care income-based tier		
Adds eligibility for underinsured to the discount care income-based tier; Additional excluded assets (e.g. car and others under Social Security Act); Extends retroactive from 180 days to 240 days; Expands presumptive eligibility with several new options like WIC and out-of-state Medicaid; Removes citizenship requirement		Adds description of family/household income
Adds underinsured to the discount care income-based tier; Additional excluded assets including primary vehicle; Extends retroactive from 180 days to 240 days; Expands presumptive eligibility (e.g., receipt of WIC		Adds description of family/household income

© 2022 Goodman C et al. *JAMA Network Open*.

and out-of-state Medicaid); Removes citizenship requirement		
		Removes coverage of rehabilitation hospital (despite ownership not changing) and includes a few additional narrow service exclusions
Increases free care income cutoff from 125% FPL to 200% FPL; Increases discounted care income cutoff from 270% FPL to 400% FPL; Includes underinsured in free care income-based tier; Extends extraordinary collection actions start time from 120 to 240 days		New state-based residency requirement (GA, NC, and SC), but underinsured restricted to GA residence only; Complex tiers of coverage based on balance; Specifies duration of six months; Changes family/household income from guarantor only to broader definition
As part of asset assessment, increases exclusion of primary vehicle from \$10k to \$15k; Increases duration from six months to one year	Narrows services to inpatient services excluding all professional services; Narrows residency eligibility to US citizens only; Excludes services for care related to criminal act, while	

© 2022 Goodman C et al. *JAMA Network Open*.

	incarcerated or in custody, self-harm; As part of asset assessment, lowers allowable home value exclusion from $250k to 125k; Adds charity care co-pay for patient “dignity.” Adds clause that the hospital can use patient information as it sees fit, and if consent withdrawn patient can be removed from program; Removes discounted care income-based tier (previously 200% FPL to 400% FPL); Removes extensive presumptive eligibility; Removes underinsured eligibility	
Increases free care income cutoff from 150% FPL to 250% FPL; Increases discounted care income cutoff from 250% FPL to 400% FPL; Removes copays; Expands presumptive eligibility	Limits coverage of undocumented immigrants to ED services only; Shortens duration from one year to six months	
Removes assets as part of eligibility for free care;		

© 2022 Goodman C et al. *JAMA Network Open*.

Expands services to include medications		
Expands presumptive eligibility		
Removes Illinois residency requirement for discounted care; Removes eligibility requirement of minimum balance of $300	Reduces discounted care income cutoff from 600% FPL to 400% FPL	Additional language on family/household income definitions
Removes Illinois residency requirement for care related to “ultra rare” diseases; Improves catastrophic coverage lowering amount collected from 25% of annual income to 20%	Removes coverage for insurance co-pays	
Increases free care income cutoff from 200% FPL to 250% FPL; Increases the discount at multiple tiers of eligibility; Improves catastrophic coverage lowering amount collected from 25% of annual income to 20%	Lowers allowable assets from equivalent of 600% FPL to 250% of FPL; Decreases duration from one year to six months	
Increases free care income cutoff from 200% FPL to 300% FPL for families; Removes $100 deposit to apply; Increases duration from		Assets section shorter and generic, removed a relatively generous exclusion of house <

© 2022 Goodman C et al. *JAMA Network Open*.

episodic to calendar year; Removes presumptive eligibility requirement that patient had clearly paid any co-pays or deductibles; Adds a section that gives specified discount to insured patients for "non-covered" services		$500k and vehicle <$50k
Adds discounted care income-based tier from 200% FPL to 250% FPL; Removes automatic exclusion for owning property other than primary residence; Removes inclusion of savings accounts in assets; Automatic 30% discount for all uninsured patients		
Adds discounted care income-based tier from 200% FPL to 400% FPL; Extends retroactive portion from six months to 12 months; Lowers eligibility requirement of minimum balance from $35 to $10	Reduces duration from six months to episodic only; "Residents outside of US" no longer eligible; Lowers free care income cutoff from 300% FPL to 200% FPL	Adds generic sections on self-pay discounts and discounts for non-covered services but leaves changes up to individual hospitals
Adds limited amount of bariatric surgery (10/year) and contraceptive services (100/year); Drops SSN	Excludes coverage for "Medicaid spenddown"	

© 2022 Goodman C et al. *JAMA Network Open*.

requirement; Removes exclusion of care related to "illegal activity;" Shortens duration of residence from six months to three months		
Increases duration from episodic to six months	Adds asset review to eligibility, must be less than equivalent of 250% FPL	
	Excludes "out of network" coverage; Disallows the option of choosing charity care rather than billing insurance	
Adds retroactive coverage of calendar year		
Extends duration from episodic to three months		Removes exclusion of "housemates" in considering income
Increases free care income cutoff from 250% FPL to 275% FPL	Changes catastrophic discount language from "may be free" to "may be 10% of family income"	
Increases free care income cutoff from 250% FPL to 275% FPL	Changes catastrophic discount language from "may be free" to "may be 10% of family income"	
		Modifies language on discount amount

© 2022 Goodman C et al. *JAMA Network Open*.

Increases free care income cutoff from 175% FPL to 200% FPL		
Expands presumptive eligibility		In asset review excludes primary vehicle, but adds requirement that liquid assets must be <$10k; Lengthier description of family income; Removes some language on citizenship requirement but will review travel visa; Specifies free care and discounted care income cutoffs at 200% FPL and 400% FPL
Adds a few exclusions of assets including a car but assets only examined if balance > $50k or "apparent significant wealth"		
Expands presumptive eligibility; Adds coverage of co-insurance payments; Excludes asset of primary vehicle from eligibility determination		Adds language to include immigrants
		Adds duration of 1 year

© 2022 Goodman C et al. *JAMA Network Open*.

Adds language about covering "out-of-network" and insured who exhausted benefits; Adds coverage for underinsured		Beaumont Medical Group added but unclear if true expansion; Self-pay discount and discounted care at incomes 200% FPL to 300% FPL changed from set rates to amounts generally billed
Increase free care income cutoff from 250% FPL to 300% FPL	Removes discounted care income tier (previously 250% FPL to 400% FPL)	
Adds discounted care tier at incomes from 250% FPL to 400% FPL; Adds language to include underinsured; Adds coverage of copays, coinsurance, deductibles, and "out of network" care		Adds asset test but with generous exclusions (e.g., up to $1 million dollar residence)
		Newly specifies assets but generous exclusions (e.g., house and vehicle <$500k and liquid assets <$300k)
Adds coverage of copay, coinsurance, deductible; Adds "out of network" for emergent care; Adds ventilators and		

© 2022 Goodman C et al. *JAMA Network Open*.

wheelchairs to equipment covered		
Removes all mention of asset tests; Reduces catastrophic coverage from balance of $100k to $75k		Adds clause that changes can be made during crises to "flex" financial assistance based on community needs
Removes exclusion of "non-resident/illegal aliens" for non-emergent care	Lowers income cutoff for presumptive eligibility from 200% FPL to 100% FPL; Adds "out of network" exclusion for non-emergent care	
Extensive addition of presumptive eligibility	Addition of service area requirement; Exclusion of any "foreign resident;" Decrease in income cutoff for free care from 250% FPL to 200% FPL; Decrease in discount cutoff from 500% FPL to 400% FPL (also makes technical change to discount calculation)	Specifies retroactive for 12 months; Removes language in assets that first $10k in assets (plus 50% of next $10k) would be excluded from consideration; Hardship discount section removed but appears it may be in a separate policy now; Adds required minimum balance of $10
		Complex changes to calculations of discounts based on income tiers

© 2022 Goodman C et al. *JAMA Network Open*.

"Healthcare crisis" section added that specifically includes coverage of COVID testing and treatment		Removed complex statement on assets
Increases discounted care income cutoff from 400% FPL to 600% FPL		
Adds special retroactive coverage for COVID19 care for uninsured		
Removes outpatient pharmacy, reference lab, and outpatient clinic exclusion from services for "category 1" group; Adds specific income cutoff for "category 2 coverage" at 300% FPL (tiered coverage based on location of service and balance)		
Removes requirement of living in service area of hospital		
Changes the discounted care income tier of 400% FPL to 600% FPL from "possible discount" to definite discount; Adds standard 35% discount above 600% FPL; Raises cutoff of $250 to $750 balance for legal action; Increases		Adds that underinsured under 400% FPL get 40% discount, prior policy did not specify discounts

© 2022 Goodman C et al. *JAMA Network Open*.

duration from three to six months		
	Excludes birth control	
	Reduces duration from six months to episodic	
Increases discounted care income cutoff from 350% FPL to 400% FPL		
Removes county-based service area requirement; Adds language about covering underinsured; Increases free care income cutoff from 250% FPL to 300% FPL; Adds presumptive eligibility for homeless and Medicaid-eligible	Disallows choosing to not run insurance in favor of charity care; Removes "upfront" 35% discount	Specifies discount percentage at discounted care income tier
Removes sentence excluding co-pay coverage; Extensive new presumptive eligibility		Changes language on assets from generic consideration of liquid/cash assets to include "non-cash" assets convertible to cash
Adds coverage of two renal transplants each year		
Removes dental services		
Allows eligibility for "foreign nationals" living in RI for >18 months		Adds language to further define "underinsured;"

© 2022 Goodman C et al. *JAMA Network Open*.

		Removes complex statement on asset test
Removes citizenship and state residency requirement	Adds "out of network" exclusion	
Increases free income cutoff from 100% FPL to 200% FPL; Increases discounted care income cutoff from 200% FPL to 400% FPL; Increases equity cutoffs for assets ($50k to $100k for house, $1k to $10k liquid); Increases duration from six months to one year; Removes small copays, appears to have been only for uninsured		Residence requirement changed from county-based service area to "catchment area" without specification; Changes service exclusions from short list of specific services to general statement that hospitals make individual determinations; Adds use of third-party tool for eligibility determinations
	Removes coverage of urology, psychiatry, and orthopedics professional services	
Increases free income cutoff 150% FPL to 250% FPL; Expands presumptive eligibility criteria		
Duration increased from episodic to six months		Free care income cutoff specified at 250% FPL; Discounted care income cutoff specified at 300%

© 2022 Goodman C et al. *JAMA Network Open*.

		FPL; Family income, asset descriptions, and service exclusions are added but prior document references other documents with these that are unavailable
Increases free care income cutoff from 200% FPL to 225% FPL		Adds exclusion of "non-covered services" for insured patients; Removes exclusion of "shortfall between government reimbursement and cost"
Extends duration from six weeks to 12 weeks	Specifies asset exclusion > $20k total and changes from liquid assets only to broader consideration of assets	
Clarifies duration of six months		
		Free care income cutoff specified at 150% FPL; Specifies that non-covered and non-allowed services under Medicaid will be considered

© 2022 Goodman C et al. *JAMA Network Open*.

		Specifies free care income cutoff at 250% FPL and discounted care income cutoff at 500% FPL
		Specifies free care income cutoff at 250% FPL and discounted care income cutoff at 500% FPL (a different hospital from above)
Free care income cutoff increased from 200% FPL to 300% FPL; Changes duration from retroactive only to six-month duration (or 12 months if fixed income); Adds catastrophic coverage section		New addition of asset test of <$50k to qualify at each level (excludes primary residence, vehicle, and retirement); Adds clause to make changes to policy during public health emergency
Removes the minimum $300 balance requirement for presumptive eligibility; Increases free care income cutoff from 200% FPL to 300% FPL; Increases discounted care income cutoff from 400% FPL to 500% FPL; Increases allowable assets from $15k to 25K		

© 2022 Goodman C et al. *JAMA Network Open*.

Improves catastrophic coverage discount amount		
		Adds clause that changes may be made to the policy during national or state emergencies
Adds discounted care income-based tier from 200% FPL to 400% FPL; Extends retroactive portion from six months to 12 months; Lowers eligibility requirement of minimum balance from $35 to $10	Reduces duration from six months to episodic only; "Residents outside of US" no longer eligible; Lowers free care income cutoff from 300% FPL to 200% FPL	Adds generic sections on self-pay discounts and discounts for non-covered services but leaves changes up to individual hospitals
Removes the residency requirements of citizenship and service area	Excludes coverage of care related to personal injury, lawsuit, and workers compensation claims	
Adds presumptive eligibility; Increases duration from episodic to six months		
Increases percentage of discounts within discounted income tier		
	Narrows residency requirement from multiple counties to single county;	

© 2022 Goodman C et al. *JAMA Network Open*.

	Excludes co-pays and deductibles	
Increases discounted care income cutoff moved from 275% FPL to 400% FPL; Removes requirement of $300 minimum balance for eligibility; Removes exclusion of "illegal aliens;" Expands presumptive eligibility		

Notes: Each row represents analysis of charity care policies for one hospital. Hospitals without available charity care policies are not shown. FPL stands for Federal Poverty Level.
